# Rates of immunization against pandemic and seasonal influenza in persons at high risk of severe influenza illness: a cross-sectional study among patients of the French *Sentinelles* general practitioners

**DOI:** 10.1186/1471-2458-13-246

**Published:** 2013-03-20

**Authors:** Ludivine Privileggio, Alessandra Falchi, Marie-Lise Grisoni, Cécile Souty, Clément Turbelin, Laure Fonteneau, Thomas Hanslik, Solen Kernéis

**Affiliations:** 1 INSERM, UMR-S 707, Paris, F-75012, France; 2 UPMC Université Paris 06, UMR-S U707, Paris, F-75012, France; 3 Laboratoire de Virologie, Université de Corse, France; 4 Département des Maladies Infectieuses, Institut de Veille Sanitaire, Saint-Maurice, France

**Keywords:** Vaccination, Influenza, General practitioners, *Sentinelles* network, Pregnancy, Obesity

## Abstract

**Background:**

Three main categories of persons are targeted by the French influenza vaccination strategy: all persons aged 65 years or over, those aged less than 65 years with certain underlying medical conditions and health care workers. The main objective of this study was to estimate rates of influenza immunization in these target groups attending a medical consultation for two consecutive influenza seasons: 2009–2010 (seasonal and pandemic vaccines) and 2010–2011 (seasonal vaccine).

**Methods:**

A standardized questionnaire was mailed to 1323 general practitioners (GPs) of the *Sentinelles* Network, collecting data on all patients seen on a randomly assigned day. For every patient, following information was collected: age, gender, BMI, presence of any medical condition that increases risk of severe influenza illness, and vaccination status for the three vaccines mentioned.

**Results:**

Two hundred and three GPs agreed to participate and included 4248 patients. Overall, in persons with high risk of severe influenza, the estimated vaccine coverages (VC) were 60%, (95% CI = 57%; 62%) for the seasonal vaccine in 2010–2011, 61% (59%; 63%) for the seasonal vaccine in 2009–2010 and 23% (21%; 25%), for the pandemic vaccine in 2009–2010. Among people aged 65 years and over (N=1259, 30%) VC was estimated for seasonal vaccines at 72% (70%; 75%) in 2010–2011 and 73% (71%; 76%) in 2009–2010, and 24% (22%; 26%) for the pandemic vaccine. The lowest seasonal VC were observed in younger persons (<65 years) with underlying medical conditions, in particular pregnant women (<10%) and overweight persons (<30%).

**Conclusions:**

Our study shows that influenza vaccination coverage among patients of the French *Sentinelles* general practitioners remains largely below the target of 75% defined by the 2004 French Public Health Law, and underscores the need for the implementation of public health interventions likely to increase vaccination uptake.

## Background

In France, seasonal influenza immunization is recommended to all persons aged ≥65 years and those with underlying medical conditions that increase their risk for influenza disease or complications [1]. Before 2010, patients with severe bronchopulmonary, cardiac, renal disease, sickle cell disease/ thalassemia, diabetes mellitus, or any immune disorder (HIV infection, immunosuppressive drugs, cancer …) were targeted by French immunization recommendations [2]. Since 2010, pregnant women (at any trimester) and overweight persons (BMI≥30) have been added to the list of patients that should receive the monovalent influenza A(H1N1) 2009 vaccine and subsequently, the seasonal trivalent vaccine yearly [3]. Every year in France, people targeted by seasonal influenza immunization receive an invitation from health authorities to get a free-of–charge vaccine, administered by their General Practitioner (GP). Despite this large national vaccination campaign, rates of immunization against seasonal influenza remain weak: the global vaccination coverage (VC) for the target population was 51% in 2007, 56% in 2008 and 57% in 2009, as estimated by the National Health Insurance [4]. To our knowledge there is no data in the literature on detailed immunization rates among at-risk populations in general practice since the influenza pandemic occurred. The objectives of this cross-sectional study were 1) to describe age distribution and risk factors of severe influenza illness in patients seen on a given day by French GPs, 2) to estimate influenza immunization rates for two consecutive seasons (2009–2010 and 2010–2011), and 3) to identify predictors of seasonal vaccination in target groups.

## Methods

### Study design

We conducted a cross-sectional study among GPs of the French *Sentinelles* Network, a computerized disease-surveillance system with active volunteer physicians located throughout mainland France, accounting for 2% of the whole population of French GPs [5]. All 1323 GPs of the network were contacted by email and invited to participate to the study in May 2011. A day of participation was randomly assigned to each GP who agreed to participate and randomization was balanced to reflect the distribution of the usual days of consultation from Monday to Saturday, as declared by participants.

### Data collection

For every patient presenting on a given day at their practice, participating GPs were asked to fill out a standardised questionnaire on age, gender, vaccination status against seasonal influenza (in 2009–2010 and 2010–2011), pandemic influenza in 2009–2010, and presence of risk factors for severe influenza illness, as defined by French health authorities (Table 1). Data on both chronic conditions and immunization status were reported by the GPs without any written confirmation. Data were obtained either by asking to the patient and/or using data from their medical records.]

**Table 1:** People targeted by influenza vaccination in 2010–2011, according to French health authorities

	
–	Age ≥ 65
–	Pregnancy at any trimester
–	Obesity (BMI ≥ 30)
–	Broncho-pulmonary disorder
–	Severe cardiac disease
–	Chronic renal disease
–	Sickle cell disease /thalassemia
–	Diabetes mellitus
–	Any immune disorder (HIV infection, immunosuppressive drugs, cancer, …)
–	Household contact of at-risk newborn

In addition, data were collected on age, gender, region of exercise for participating GPs. Regions of exercise were categorised in rural or urban areas according to the mean number of inhabitants (urban municipalities are municipalities without continuous built area of 2000 inhabitants, and those with less than half of the municipal population is in an area of continuous built, as defined by the French Institute of Demography [6]). GPs participating to the study were compared to the whole population of French GPs, based on national data [7] on age, gender, geographical area and type of practice (group or single medical practice).

### Statistical analysis and sample size calculations

Previous studies of the *Sentinelles* Network estimated that on a normal day of consultation, GPs would see an average of 20 patients [8]. With a response rate of 20%, we calculated that we could include approximately 5,300 patients and estimate rates of immunization as low as 30% with an accuracy of 5%.

Results are expressed as the median [Interquartile Range] for continuous variables and N (%) for categorical variables. Immunization rates are given with their 95% confidence intervals (95%CI). Predictors of immunization against seasonal influenza in 2010–2011 were studied by fitting generalized linear models with random intercept to the data. Vaccination against influenza in 2010–2011 was the response variable and ‘GP’ effect was regarded as a random effect. Variables analysed in the univariate analysis were: age, gender, vaccination against seasonal influenza in 2009–2010, vaccination against pandemic influenza in 2009–2010, and number of individual risk factors for severe influenza illness (taken as <1 or ≥2). Factors achieving a *p*-value < 0.20 in the univariate analysis were included in the multivariate analysis. A backward stepwise variable selection procedure was used to remove factors with *p*-value > 0.05. Statistical analysis was performed using the R software version 2.13 (R Development Core Team, R Foundation for Statistical Computing, Vienna, Austria, http://www.r-project.org).

### Ethics

The protocol was conducted in agreement with the Helsinki declaration. The protocol was approved by the ethical committee (CPP Ile de France V). We obtained authorization from the French Data Protection Agency (CNIL, registration number #471393) and all data transmitted for statistical analysis were anonymous. Verbal non-opposition was obtained. If a child was selected, non-opposition for participation in the study was requested from a parent (or legal guardian).

## Results

### Practitioners

Of 1,323 GPs of the *Sentinelles* Network solicited by email in May 2011, 262 agreed to participate (20%), and 203 (77%) returned their questionnaire. From June to September 2011, 203 GPs included 4248 patients.

One hundred and seventy four GPs (86%) were male. Their median age was 55 [Interquartile Range, IQR = 51–59] at the time of the study, and most (N=160, 79%) were aged >50 years. All administrative regions of metropolitan France were represented. One hundred and thirty three (67%) GPs were working in rural areas, and 86 (52%) belonged to group medical practices. On day of participation, each GP had included 20 patients (median, IQR = 15–25). Participating GPs were comparable to the whole population of French GPs with respect to age (mean age 52 years in both, Student test p-value, *p*= 0.86), areas and type of practice but there was a higher proportion of men in our sample (86% and 71% respectively, Chi-2 test p-value *<*0.0001).

### Patients

Demographical and clinical data of patients are displayed in Table 2. Most patients were women (56%) and their median age was 50 [IQR= 27–68]. Of 4,248 patients included, 1,827 (43%) were targeted by influenza vaccination, either because of age (≥65, N=1259) or the presence of an individual risk factor of severe influenza disease (N=568) (Table 2). The most represented individual risk factor was obesity (N=573, 14%), followed by diabetes mellitus (N=361, 9%), severe cardiac disease (N=281, 7%), and chronic bronchopulmonary diseases (N=243, 6%). Other risk factors were less represented: 5 (<1%) patients had chronic renal disease and 44 women were pregnant. Around 2% (N=64) of study patients had chronic immune suppression, either due to HIV infection (N=22, <1%) or other medical conditions such as active solid cancer (N = 23), long term corticosteroid therapy and/or other immunosuppressive drugs for autoimmune diseases (N = 13), malignant hemopathies (N = 9), chronic liver diseases (N = 5), or solid organ transplantation (N = 2).

**Table 2:** Characteristics of patients included, N(%) or median (min -max)

	**Patients included (N=4248)**
Female	2373 (56%)
Age in years	50 (<1 – 103)
Age ≥ 65	1259 (30%)
Age < 65 and at least one individual risk factor	568 (13%)
Pregnancy	44 (1%)
Obesity	317 (7%)
Chronic bronchopulmonary disease	117 (3%)
Severe cardiac disease	48 (1%)
Chronic renal disease	5 (<1%)
Sickle cells/ thalassemia	2 (<1%)
Diabetes mellitus	129 (3%)
HIV infection	18 (<1%)
Other immunosupression	24 (<1%)
Total patients targeted by vaccine recommendations	1827 (43%)

### Seasonal vaccine

**Figure 1:**
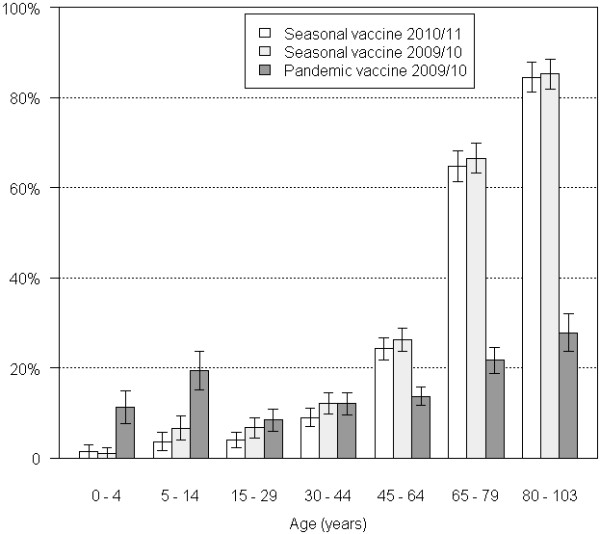
**Rates of immunization by age group.** Bars present the rates of immunization for three influenza vaccines (seasonal 2009–2010 seasonal 2010–2011 and pandemic), and their 95%CI.

Overall rates of immunization against seasonal influenza were 32%, (95%CI: 30%; 33%) in 2009–2010 and 30% (95%CI: 28%; 31%) in 2010–2011 (chi-square p-value = 0.06, Table 3). Age distributions were not statistically different between the two seasons (p =0.06) (Figure 1). Rates of immunization in patients aged ≥ 65 were 72% (95%CI: 60%; 81%) in 2009–2010 and 73%, (95%CI: 62%; 81%) in 2010–2011 (p-value = 0.47). For both seasons, the highest rates of immunization against seasonal influenza were observed in adults aged ≥65 with at least one underlying medical condition, ranging from 75% to 100% depending on the risk factor.

**Table 3:** Vaccination coverage in % (95%CI) for three influenza vaccines

	**Seasonal vaccine 2010-2011**	**Seasonal vaccine 2009-2010**	**Pandemic vaccine 2009-2010**
Patients targeted by vaccine recommendations (N=1827)	60 (57–62)	61 (59–63)	23 (21–25)
Age ≥ 65 (N=1259)	72 (70–75)	73 (71–76)	24 (22–26)
Obesity (N=256)	75 (70–80)	76 (71–81)	29 (24–35)
Severe cardiac disease (N=233)	83 (78–88)	85 (81–90)	32 (26–38)
Diabetes mellitus(N=232)	81 (76–86)	81 (76–86)	35 (28–41)
Chronic bronchopulmonary disease (N=126)	87 (81–93)	87 (81–93)	32 (23–40)
Chronic renal disease (N=31)	90 (80–100)	90 (80–100)	23 (8–39)
HIV infection (N=4)	100 (100–100)	75 (33–100)	67 (13–100)
Other immunosupression (N=18)	78 (59–97)	78 (59–97)	22 (3–41)
Age < 65 and ≥ 1 individual risk factor (N=568)	32 (28–36)	34 (30–38)	21 (17–24)
Obesity (N=317)	25 (20–30)	27 (22–32)	18 (14–22)
Severe cardiac disease (N=48)	43 (28–57)	47 (33–61)	32 (19–45)
Diabetes mellitus(N=129)	53 (45–62)	57 (49–66)	25 (17–32)
Chronic bronchopulmonary disease (N=117)	41 (32–50)	44 (35–53)	24 (17–32)
Chronic renal disease (N=5)	75 (33–100)	75 (33–100)	50 (1–99)
HIV infection (N=18)	67 (45–88)	61 (39–84)	33 (12–55)
Other immunosupression (N=24)	52 (32–73)	57 (36–77)	30 (12–49)
Pregnancy (N=44)	5 (0–11)	7 (0–14)	7 (0–15)
Household contact of at-risk newborn(N=46)	37 (23–52)*	30 (17–44)*	21 (9–34)*

*Missing data: 812.

Conversely, VC were much lower in <65 at-risk adults. Only 34% (95%CI: 30%; 38%) and 32% (95%CI: 28%; 36%) had been vaccinated against seasonal influenza in 2009–2010 and 2010–2011 respectively. In the three most represented risk factors among patients aged <65 (obesity, chronic bronchopulmonary disease and diabetes mellitus), VC hardly reached 53-57% in diabetic patients, 41-44% in those with pulmonary disorder, and less than 30% in overweight persons. In patients with chronic immune suppression, rates of immunization were slightly higher: 52-67% (N=42). On the other hand, less than 5% of the forty four pregnant women were vaccinated.

In the univariate analysis, factors associated with immunization against seasonal influenza in 2010–2011 in target groups were: to have received seasonal influenza vaccine in 2009–2010 (OR=220.0, 95%CI: 131.7; 369.8), to have received pandemic influenza vaccine in 2009–2010 (OR=9.9, 95%CI: 6.9; 14.4) and to have ≥2 individual risk factors for severe influenza illness (OR=1.8, 95%CI: 1.4; 2.4). These three predictors remained significantly associated with immunization in 2010–2011 in the multivariate analysis (OR = 321.4 (95%CI: 221.7; 465.9) ; 1.9 (95%CI: 1.3; 2.7) and 2.9 (95%CI: 1.7; 4.7) respectively).

### Pandemic vaccine

The overall pandemic VC was estimated at 16% (95%CI: 15%; 17%) in our sample and reached 23% (95%CI: 21%; 25%) in individuals with risk factors for severe influenza illness. Rates of immunization were higher in children aged <15 than that observed for seasonal vaccines (p-value<10^-5^ for both vaccines), reaching 11% (95%CI: 8%; 15%) in the 0–5 years and 19% (95%CI: 15%; 24%) in the 5–14 years (Figure 1). However, as compared to seasonal vaccines, pandemic immunization rates in adults aged ≥65 years as well as those <65 with one risk factor were low. For example in patients aged >65 with cardiac disease, VC were of 32% (95%CI: 26%; 38%) for pandemic vaccine as compared to 85% (95%CI: 81%; 90%) for the seasonal vaccine on the same season.

## Discussion

Our estimates of VC against seasonal and pandemic influenza were generally comparable or higher than that previously reported in certain categories of patients, such as those aged 65 and over, or with chronic cardiac, pulmonary diseases or diabetes mellitus.

Our estimates of VC for the 2010–2011 season were comparable to those estimated by the French National Health Insurance for subjects below 65 years with chronic conditions; but higher for persons aged 65 years and over [9], as shown in Table 4. In patients aged ≥65, who accounted for around one third of our sample, VC were above 70% for the influenza seasonal vaccines for both seasons, similar (76%) to that reported in another study among patients of the French GPs in 2006–2007 season [10], but substantially higher than VC value (61%) estimated in general population aged 65 and over in France in 2010–2011 season [11], in Spain in 2010–2011 (59%) and 2009–2010 (63%) [12], and in the United States in 2009–2010 (69%) [13]. In our sample of cardiovascular disease sufferers, VC varied notably between the two age groups: 43-47% below the age of 65, and 83-85% above 65. In comparison, in Spain, in 2003, vaccination coverages were of 17% in people aged 16–49, 34% in those aged 50–64 and 69% in patients aged 65 years and over [14]. In the USA, these proportions for the 1999–2000 influenza season were of 23% (95% CI, 18%–27%), 49% (95% CI, 44%–54%), and 77% (95% CI, 74%–80%) respectively [15]. Similar features were observed for patients with chronic respiratory diseases: whereas VC were above 85% in those aged 65 and over, younger patients were less likely to be vaccinated (VC: 41-44%), although chronic bronchopathy is a well-documented risk factor for severe influenza. A Spanish study showed that influenza vaccination coverage among subjects with chronic respiratory diseases in 1993, 1995–1997 and 2001 were 45%, 46% and 44%, respectively and variables that increased the likelihood of having been vaccinated were: higher age, presence of another concomitant chronic disease, poor perception of health, non-smoker status, and being married [16,17]. Yet, another Spanish study was conducted in early 2003 in population over 40 years of age with Chronic Obstructive Pulmonary Disease and treated at primary-care centers. Over 10 000 patients, 87% reported having been vaccinated in the most recent influenza campaign, therefore a rate comparable to that found in our study in the ≥65 only. Beside socio-demographic data, health-status related variables, and lifestyles analysis, authors found that the factor that was most strongly associated with influenza vaccination was a precedent vaccination against pneumococcal infection [18]. Similarly, in diabetic patients, we report seasonal vaccine coverages of 53-57% in <65 and 81% in ≥65 patients, whereas in Spanish diabetic adults, VC were estimated at 57% in 2003 [19] and only 34% among those aged <65 years in 2009–2010 [20].

**Table 4:** Vaccination coverages in % (95% CI) for seasonal influenza vaccine in France in 2010/2011, as estimated in three different studies

**Methodology**	**Origin of data**	**VC for seasonal influenza vaccine in 2010/2011 (95% CI)**	
		**6m-64yo with chronic illness**	**≥65yo**
Administrative data	CNAMTS administrative data (general population) [9]	33.1%	54.0%
Cross-sectional national telephone survey	French Institute for public health surveillance's survey (general population) [11]	46.6% (39.7-53.6)	61.0% (56.7-65.0)
Standardized survey on a randomly assigned single day in general practice	Patients of GPs of the French *Sentinelles* Network (present study)	32% (28–36)	72% (70–75)

Overall rate of immunization against pandemic influenza A(H1N1)2009 was very low in our sample (16%, 95%CI: 15%; 17%), higher than national estimates in the general population (<8%) [21], but close to that (17%) reported in another French cross-sectional study conducted among GPs in spring 2010 (the MOTIVAC study) [22]. These results likely reflect the public perception of low risk from the disease and the negative impact of controversies concerning the safety and quality of the pandemic vaccines [23,24]. Distinction must be made between GP involvement in a pandemic context and GP involvement in a seasonal context. Indeed, in France in the context of the H1N1 pandemic, their role was relegated to second place in favor of mass vaccination in health centers open for the event. This campaign was considered as a failure because a very small part of the population joined the vaccination while logistical and financial efforts to organize it were particularly important. Hence the importance of the involvement of the specialists in the promotion of vaccination in patients with specific risk factors (such as pregnancy, severe pulmonary, cardiac and renal diseases).

In our study population, 14% of patients were obese, and 11% in those aged <65. More than 50% of them had no other risk factor for severe influenza. Obese poorly vaccinated were those under <65 years for seasonal flu and all subjects of this sample for pandemic influenza. It must be noted that there is no data in the literature on the obese influenza vaccination coverage. Sample sizes were low in other target groups, thus estimates of VC should be interpreted with caution in these categories. Forty four pregnant women could be evaluated for both influenza seasons in our study population. VC for seasonal and pandemic influenza vaccines were particularly low (5-7%). In the United States, were influenza vaccination is routinely recommended in all pregnant women, VC was estimated at 51% for seasonal influenza vaccine and 47% for the pandemic influenza vaccine in 2009–2010 [25]. Similarly, a large number of pregnant women in the Netherlands reported to be vaccinated against 2009 influenza A (H1N1): 63% (2993 respondents/ 14529 patients invited to complete an internet survey) [26]. Influenza vaccination of pregnant women was a focus of public health efforts during the 2009–10 season in France. Before this season, vaccine recommendations didn’t apply to them if they had no identified risk factor for severe influenza. However, the VC reported here suggest a poor adherence to influenza vaccination in this category, as confirmed in another large cohort of French pregnant women in 2009 [27], were 29% of women reported to be vaccinated against pandemic influenza. It might be related to the physicians and patients insufficient information about the safety of the pandemic vaccine [28].

In the 18 patients living with HIV aged 65 or less, 61-67% declared being vaccinated against seasonal and 33% against pandemic 2009 A/H1N1 influenza. In the US influenza vaccination coverage in HIV-Infected patients increased from 29% in the 1990 to 42% in the 2002 season [29]. A French study reported comparable A/H1N1 vaccine coverage (44%) among HIV-infected patients [30]. The analysis of determinants in the target groups showed that VC for seasonal influenza in 2010–2011 was related to previous vaccinations and the number of risk factors for severe influenza illnesses. This significant association between vaccine uptakes was shown previously [31].

Our study has both strengths and limitation that warrant discussion. First, the rate of participation (20%) was slightly lower to those reported in previous studies of the *Sentinelles* network [32]. Probably, the main reason was that participating GPs were asked to fill the questionnaire for all patients presenting at their practice on a given day, which represented an important effort. In addition, summer holidays covered a large part of the data collection period. However, besides response rate, comparison of respondents to the whole population of Sentinel GPs did not show statistical differences neither on on socio-demographic data nor on type of practice. Second, general applicability of our results might be limited by the fact that the study was conducted in the specific population of French *Sentinelles* GPs. Comparison of our population to the whole population of French GPs did not show major differences neither on socio-demographic data nor on type of practice. However, the *Sentinelles* network is composed by volunteer GPs who collect real-time epidemiological data, suggesting a specific interest for public health, in particular for immunization. This limitation could explain the high vaccination rates observed, as compared to studies in general population. Third, since participation rate was relatively low, some target groups were less represented, leading to wide CI for rare target groups, in particular for HIV infection, other immunosupression, chronic renal disease and pregnancy. Patients of these categories probably get medical care from specialists of their chronic disease, hence underlining the importance of conducting specific studies in specialized medical services. And finally, immunization data were self-reported by the patient and/or the GPs and not written-confirmed, leading to some degree of imprecision in evaluation of the immunization status.

Despite these limitations, such studies among *Sentinelles* GPs can be complementary to the population-based studies. Indeed, our study allowed to collect data rapidly, on a large number of patients, and was resource-saving since all GPs of the Sentinel Network could easily and simulatenously be contacted via an automatic-generated email. If necessary, such study could be renewed yearly to follow variations of the VC from on year to another, for example in case of occurrence of an influenza pandemic. We also specifically described the characteristics of patients attending a medical consultation with French GPs, therefore it could help identify populations that could be counselled by their GPs to get vaccinated. In addition, such design might limit recall biases compared to telephone surveys. Indeed, estimation of VC was based both on answers of patients and on data collected by the GPs in their medical records. Usually, participation biases are in favor of better vaccinated individuals, leading to some over-estimation of coverage in the telephone surveys.

The prevalence of chronic conditions in our study sample was comparable to that estimated in the general population regarding data published by the French Ministry of Health [33] for pregnancy, obesity, diabetes mellitus, Sida-infection and chronic cardiac diseases (prevalence estimates of 1%, 13% 4%, 0.05% and 1% respectively in the general population). Persons aged 65 and over were over-represented in our sample, whereas prevalence of chronic bronchopulmonary and renal diseases were low, maybe due to underdiagnosis. This confirms that for most chronic conditions, involvement of GPs can have a potentially important impact to improve VC. On the other hand, for patients with chronic bronchopulmonary and renal diseases, vaccination promotion limited to GP could be less efficient and specialists must be involved.

## Conclusions

This study gives an overview of characteristics of patients seen on a given day in French general practices, as well as estimates of rates of immunization against both seasonal and pandemic influenza in target groups. Our estimates of VC are generally higher than that reported by others in the general population. GPs receive a very large proportion of patients targeted by vaccine recommendations (great variety of chronic underlying conditions and elderly persons). In addition, a lot of these patients are not regularly followed by other physicians who could offer vaccination (patients with obesity, diabetes…). We also report that pandemic influenza VC were lower than seasonal VC in all categories of patients. Contrary to a standard seasonal vaccination campaign, the administration of pandemic vaccine in 2009 in France was not primarily assured by GPs. This underlines the crucial role of GPs in promoting immunization in the community. There are some examples of successful public health measures through GP. Their involvement in colorectal cancer mass screening allows identification of high risk people who can then be managed with a more suitable screening protocol [34]. This study underlines the need to consider the role to be given to GPs in the choice and implementation of immunization strategies.

## Competing interests

The authors declare that they have no competing interests.

## Authors’ contributions

LP conceived the design of the study, participated in acquisition and interpretation of data, and drafted the manuscript. AF participated in the design of the study, interpretation of data and helped to draft the manuscript. MLG participated in the design of the study, interpretation of data and statistical analysis. CS participated in the interpretation of data and drafting of the manuscript. CT participated in the design of the study, provided technical assistance on acquisition of data and helped to draft the manuscript. LF participated in the interpretation of data and drafting of the manuscript. TH participated in the design of the study, interpretation of data and helped to draft the manuscript. SK participated in the design of the study and interpretation of data, performed statistical analysis and helped to draft the manuscript. All authors read and approved the final manuscript.

## Pre-publication history

The pre-publication history for this paper can be accessed here:

http://www.biomedcentral.com/1471-2458/13/246/prepub
